# Chemoinformatics Studies on a Series of Imidazoles as Cruzain Inhibitors

**DOI:** 10.3390/biom11040579

**Published:** 2021-04-15

**Authors:** Alex R. Medeiros, Leonardo L. G. Ferreira, Mariana L. de Souza, Celso de Oliveira Rezende Junior, Rocío Marisol Espinoza-Chávez, Luiz Carlos Dias, Adriano D. Andricopulo

**Affiliations:** 1 Laboratório de Química Medicinal e Computacional, Centro de Pesquisa e Inovação em Biodiversidade e Fármacos, Instituto de Física de São Carlos, Universidade de São Paulo, Av. João Dagnone 1100, São Carlos, SP 13563-120, Brazil; alex.medeiros@usp.br (A.R.M.); leonardo@ifsc.usp.br (L.L.G.F.); marianalaureano@gmail.com (M.L.d.S.); 2 Instituto de Química, Universidade Estadual de Campinas, Campinas, SP 13084-971, Brazil; celso@ufu.br (C.d.O.R.J.); romaes.90@gmail.com (R.M.E.-C.); ldias@unicamp.br (L.C.D.)

**Keywords:** cruzain, QSAR, Chagas disease, inhibitor, LBDD, SBDD

## Abstract

Natural products based on imidazole scaffolds have inspired the discovery of a wide variety of bioactive compounds. Herein, a series of imidazoles that act as competitive and potent cruzain inhibitors was investigated using a combination of ligand- and structure-based drug design strategies. Quantitative structure–activity relationships (QSARs) were generated along with the investigation of enzyme–inhibitor molecular interactions. Predictive hologram QSAR (HQSAR, *r*^2^_pred_ = 0.80) and AutoQSAR (*q*^2^ = 0.90) models were built, and key structural properties that underpin cruzain inhibition were identified. Moreover, comparative molecular field analysis (CoMFA, *r*^2^_pred_ = 0.81) and comparative molecular similarity indices analysis (CoMSIA, *r*^2^_pred_ = 0.73) revealed 3D molecular features that strongly affect the activity of the inhibitors. These findings were examined along with molecular docking studies and were highly compatible with the intermolecular contacts that take place between cruzain and the inhibitors. The results gathered herein revealed the main factors that determine the activity of the imidazoles studied and provide novel knowledge for the design of improved cruzain inhibitors.

## 1. Introduction

Neglected tropical diseases (NTDs) affect more than 1.5 billion people worldwide in more than 150 countries, mainly in developing nations [1]. Among NTDs, Chagas disease is endemic in 21 Latin American countries and affects 6 to 7 million people worldwide [2]. The highest burden in a nonendemic country occurs in the USA, where approximately 300,000 people have the disease [3]. Chagas disease severely impacts the economy of endemic countries, causing losses of over US $7.2 billion annually and a burden of 243,600 disability-adjusted life years (DALYs) [4,5]. The causative agent of the disease, the protozoan *Trypanosoma cruzi*, is transmitted mainly through the feces of hematophagous triatomines known as kissing bugs [3]. Disease dissemination can additionally occur by blood transfusion, organ transplantation, the ingestion of infected food, and transplacental transmission. The etiological treatment of Chagas disease has not improved since the 1970s and still relies on nifurtimox and benznidazole, two nitroheterocyclic drugs that lack efficacy in the chronic phase of the disease and cause serious adverse effects [6]. These drawbacks combined with the emergence of drug resistance reveal the need for new, safe, and effective drugs for the treatment of Chagas disease.

In this study, the molecular features that determine the activity of a series of imidazole-based cruzain inhibitors were investigated. The imidazole ring is broadly present in natural products from a diversity of sources and is a key structural motif of essential biomolecules such as nucleic acids, purine, histamine, and histidine [7,8]. Moreover, imidazole-containing compounds feature relevant biological activities such as antifungal, anti-inflammatory, antidiabetic, antibacterial, antiviral, antiparasitic, and anticancer activities [7,8,9,10].

Cruzain is the main cysteine protease from *Trypanosoma cruzi* and is a validated molecular target for Chagas disease drug discovery. The enzyme is vital for the parasite physiological processes such as cell replication, nutrition, the invasion of host cells, and the evasion of the host immune system [11,12]. Following these findings, multiple classes of inhibitors, including imidazoles, benzimidazoles, cyclic imides, and vinyl heterocycles, among others, have been designed as cruzain inhibitors [13,14,15,16].

In this study, four quantitative structure–activity relationship (QSAR) methods were used: The 2D tools hologram QSAR (HQSAR) and AutoQSAR and the 3D methods comparative molecular field analysis (CoMFA) and comparative molecular similarity indices analysis (CoMSIA) [17,18,19,20]. Statistically sound and predictive QSAR models were derived for all the techniques. Molecular docking was used to align the compounds structurally and assess their binding conformations within the active site of cruzain. The integrated analysis of the QSAR and molecular docking studies yielded novel information on the molecular features that drive cruzain inhibition.

## 2. Materials and Methods

### 2.1. QSAR and Molecular Modeling Tools

The HQSAR, AutoQSAR, CoMFA, and CoMSIA models were built as previously described [21,22,23,24] using SYBYL-X 2.1 (Certara Inc., Princeton, NJ, USA) and Maestro (release 2016-3) (Schrӧdinger LLC, New York, NY, USA). The 3D structures of the compounds were built using Epik at pH 5.5 and minimized using LigPrep and OPLS3 (Schrӧdinger LLC, New York, NY, USA) [25,26,27]. The X-ray structure of cruzain deposited in the Protein Data Bank (PDB 3KKU, 1.28 Å) [28] and GOLD 5.3 (Cambridge Crystallographic Data Centre, Cambridge, UK) [29] were used in the molecular docking studies. The enzyme-inhibitor complexes were visualized using Maestro (release 2016-3) and Chimera (University of California, San Francisco, CA, USA) [30].

### 2.2. Dataset

**Figure 1:**
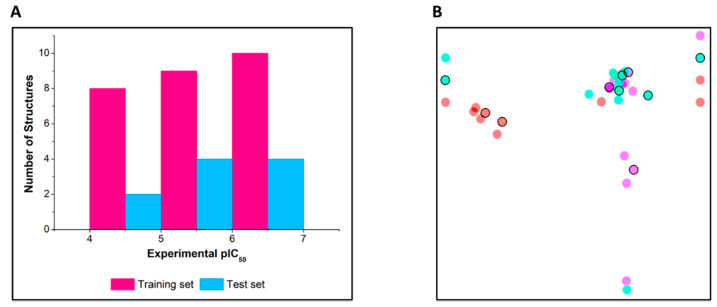
(**A**) Distribution of the dataset into training and test sets according to pIC_50_ ranges. (**B**) Structural similarity map. The encircled points represent test set compounds. Structural similarity is inversely proportional to the distance between the points, and the colors represent pIC_50_ intervals. Red: 4.00–5.06; cyan: 5.07–6.12; and magenta: 6.13–6.92.

The dataset used to build the QSAR models consists of 37 compounds (Table 1) that were designed and tested against cruzain in our research group. The series was designed based on the structure of lead compound 1, which is a competitive cruzain inhibitor identified in a virtual screening effort [13]. The IC_50_ values (concentration of inhibitor required to inhibit 50% of enzyme activity) determined against cruzain were converted into pIC_50_ (negative logarithm of IC_50_) to properly scale the data for the QSAR studies (pIC_50_ values ranging from 4.00 to 6.92). Importantly, the dataset compounds have the same mechanism of action against cruzain, namely, competitive inhibition, and the IC_50_ values were determined under the same experimental conditions. The dataset compounds were divided randomly into 27 training set molecules for the development of the QSAR models and 10 test set compounds for external validation. This selection was automatically performed by AutoQSAR with the generation of 497 physicochemical and topological descriptors and different types of molecular fingerprints (linear, radial, dendritic, and molprint2D) [17,31]. The same training and test sets were used to derive all QSAR models. Figure 1A shows that the algorithm was able to select compounds so that inhibitors across a broad range of pIC_50_ values were included in both the training and test sets. To visualize the structural distribution of the dataset, a principal component analysis (PCA) was conducted [32]. The similarity map generated by the PCA routine was built using UNITY fingerprints to assess the structural similarity between the compounds [33]. The PCA algorithm derived two principal components that were used as the initial coordinates to plot the similarity map. All points in the map were plotted by calculating the Tanimoto distances between the UNITY fingerprints [34]. The structure similarity map is shown in Figure 1B, in which each point represents a compound. The distance between the points is inversely proportional to structural similarity, and the colors represent different pIC_50_ intervals.

### 2.3. Molecular Docking

**Figure 2:**
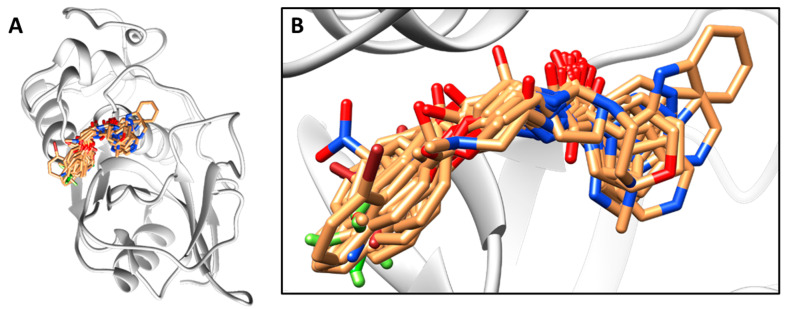
Cruzain inhibitors aligned within the binding site of the X-ray structure of cruzain (PDB 3KKU, resolution of 1.28 Å). (**A**) View of the entire 3D structure of cruzain with the aligned compounds. (**B**) Magnification of the active site of the enzyme and aligned compounds. The structure of cruzain is depicted as a cartoon, and the inhibitors are represented as sticks.

Molecular docking was performed using the X-ray structure of cruzain (PDB 3KKU, 1.28 Å) [28] and GOLD 5.3 [29]. The structure of cruzain was prepared by adding hydrogen atoms and excluding solvent and the co-crystallized inhibitor. The binding site was defined as a 10-Å sphere centered on the Cys25 sulfur atom. All the binding site residues were checked for possibly flipped orientations, tautomerism, and protonation states. Cys25 was kept negatively charged, and His162 was kept protonated. The binding site was restricted to the solvent-accessible surface by applying the cavity detection routine. The genetic algorithm was set for a search efficiency of 200%. GoldScore was used as the scoring function, and the best conformation for each inhibitor was used in the 3D QSAR modeling. Figure 2 depicts the inhibitors aligned in the active site of cruzain.

### 2.4. QSAR Modeling

Only the training set was used in the production and internal validation of all QSAR models. The best models were evaluated for their external predictive ability by deriving the predictive correlation coefficient (*r*^2^_pred_) for the test set compounds. The HQSAR models were initially derived using fragments containing 4 to 7 atoms and the default hologram lengths: 53, 59, 61, 71, 83, 97, 151, 199, 257, 307, 353, and 401 bins. Atom type (A), bond type (B), hydrogen atoms (H), connectivity (C), and chirality (C) were combined and used as fragment distinction criteria. Models were generated using partial least squares (PLS) regression, and the optimum number of components was determined using the leave-one-out (LOO) cross-validation procedure, which derived the cross-validated correlation coefficient (*q*^2^). Then, the optimum number of components was used to determine the full non-cross-validated correlation coefficient (*r*^2^) for the entire training set. Progressive scrambling was used to estimate the susceptibility of the models to chance correlations.

The AutoQSAR models were built using linear, dendritic, radial, and molprint2D fingerprints along with 497 physicochemical and topological descriptors [35]. To reduce the level of redundancy among descriptors, the feature selection routine was used to select an optimized nonredundant descriptor subgroup. Then, the default machine-learning techniques were applied to different combinations of descriptors and fingerprints to generate models based on random splits of training and test sets. Next, the robustness of each model was assessed by a score value that accounts for predictive accuracy regarding both the training and test sets. The proportion of the training set with respect to the entire dataset was varied from 70 to 80%.

CoMFA steric and electrostatic interaction energies between the aligned compounds and an sp^3^ hybridized carbon atom (charge = +1) were computed using Coulomb and Lennard–Jones potentials, respectively [19]. The default CoMFA scaling and a cutoff value of 30 kcal/mol were used to generate the interaction fields. The 3D cubic lattice that encloses the compounds was generated using grid spacings from 0.5 to 2.0 Å. The region-focusing procedure was applied to refine the models and increase the resolution of the CoMFA contour maps. The contour maps were built by computing the pairwise products between the standard deviations and PLS coefficients of the CoMFA columns (StDev*Coeff) using the region-focusing method [19].

CoMSIA similarity indices were computed by taking the distance between the atoms of the compounds and the virtual probe. The dependence of the similarity indices on the distance between the probe and the atoms was assessed by the default Gaussian function [20]. The structural alignment illustrated in Figure 2 was used to run the CoMSIA analyses. The 3D cubic lattices were generated using grid spacings from 0.5 to 2.0 Å. The resolution of the CoMSIA contour maps was increased by applying the region-focusing method. The attenuation factor of the Gaussian function was kept at its default value of 0.3. The CoMSIA contour maps were derived by using the StDev*Coeff method.

### 2.5. Applicabilty Domain

The definition of the applicability domain is one of the principles established by the Organization for Economic Co-operation and Development (OECD) to assess the validity of QSAR models. By applying the applicability domain concept, one can evaluate the uncertainty in predicting the target variable for a molecule considering its similarity to the compounds that were used to construct the QSAR model [36]. The applicability domain plots for the QSAR models generated in this work were built using the influence graph (leverage) versus Student residues (activity residues) approach. The applicability domains for each final QSAR model were built using the Chemoface program [37].

## 3. Results and Discussion

### 3.1. HQSAR and AutoQSAR Models

The initial HQSAR models were developed using all available hologram lengths (53 to 401 bins) and fragment sizes varying from 4 to 7 atoms. The following combinations of fragment distinction parameters were used to generate the molecular holograms: A/B/C, A/B/C/Ch, A/B/C/H, and A/B/C/H/Ch. The results for cross-validated LOO and full models are shown in Table 2. Since significant results were obtained for all models, we evaluated the leverage of fragment size on the statistical consistency while keeping the same fragment distinction criteria. The best models obtained with this procedure are listed in Table 3. Additionally, Table 3 shows the *r*^2^_pred_ values, which confirm the predictive ability of these models for the test set.

**Figure 3:**
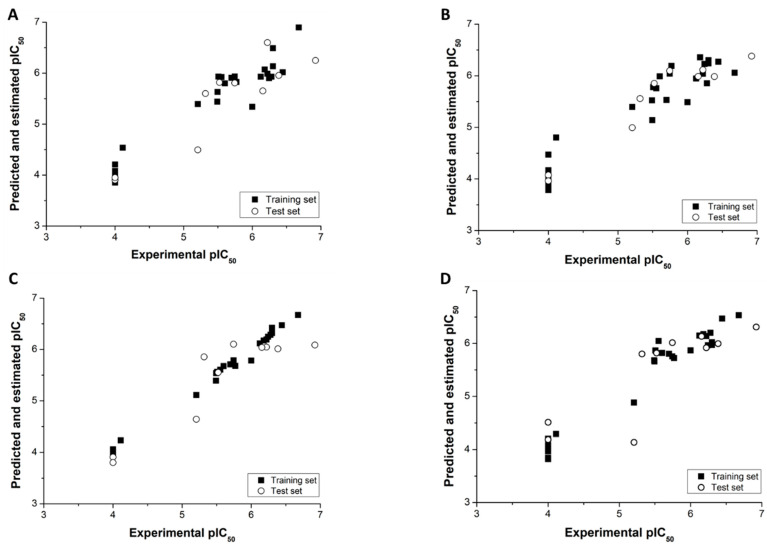
Experimental and predicted pIC_50_ for the final QSAR models. (**A**) HQSAR; (**B**) AutoQSAR; (**C**) CoMFA; and (**D**) CoMSIA. Solid squares represent the training set, and open circles represent the test set.

Table 3 shows that variation in fragment size slightly improved the key statistical indicator *q*^2^. Among the five HQSAR analyses in Table 3, model 6 exhibited the most significant results (*q*^2^ = 0.71 and *r*^2^_pred_ = 0.80). To assess the susceptibility of model 6 to chance correlations, a Y-randomization test was carried out. The noise introduced in the model by systematic perturbations in the dependent variable caused an expected drop in the *q*^2^ value. The randomized model had a *Q*^2^ = 0.42, which attests to the stability of the original QSAR analysis against chance correlations. Stable models should, additionally, yield progressive scrambling effective slopes close to unity, which is the case for model 6 (*dq^2^/dr*^2^_yy_ = 1.01). In addition to the procedures for internal validation, model 6 was assessed for its external predictive ability for the test set compounds. The good agreement between the experimental and predicted pIC_50_ values along with an *r*^2^_pred_ of 0.80 demonstrate the high predictive power of the best HQSAR model for novel, structurally related compounds. Experimental and predicted pIC_50_ values for the complete dataset are listed in Table 4 and depicted in Figure 3.

The AutoQSAR models were built using all available molecular fingerprints—linear, radial, molprint2D and dendritic—and 497 physicochemical and topological descriptors. Optimized combinations of a nonredundant descriptor subset and fingerprinting methods were correlated with the biological activity using the default machine learning algorithms of AutoQSAR. The statistical indicators for the best models are presented in Table 5.

All models in Table 5 performed well regarding both the training and test sets. The model with 72% of the compounds in the training set and dendritic fingerprint exhibited the best performance, as demonstrated by the highest score value of 0.90. Similarly, considering only the training set, this model yielded the best coefficient of determination (*r*^2^ = 0.89). Considering only the test set, the model with a training set of 80% performed slightly better than the other models (*q*^2^ = 0.91). Table 4 and Figure 3B demonstrate the suitable agreement between the experimental and predicted pIC_50_ values and the high predictive power of the dendritic AutoQSAR model.

### 3.2. CoMFA and CoMSIA Models

CoMFA and CoMSIA models were built using aligned cruzain inhibitors, as illustrated in Figure 2. Region focusing weighted by StDev*Coeff values from 0.3 to 0.9 and grid spacings from 0.5 to 2.0 Å were applied to derive the PLS regressions. Table 6 shows the statistical indicators for the best 3D QSAR models. Grid spacings of 1.1 and 1.3 Å for CoMFA and CoMSIA, respectively, and a StDev*Coeff value of 0.3 produced the most statistically sound models. The PLS-LOO regression models produced *q*^2^ values of 0.72 and 0.63 for the best CoMFA and CoMSIA analyses, respectively. Additionally, the best CoMFA and CoMSIA models exhibited *r*^2^_pred_ values of 0.81 and 0.73, respectively, demonstrating the predictive ability of these models for the test set. Progressive scrambling generated critical slopes (*dq*^2^/*dr*^2^_yy_) of 1.09 for CoMFA and 1.10 for CoMSIA. *Q*^2^ values of 0.52 and 0.48 were obtained for the CoMFA and CoMSIA scrambling analyses, respectively. Table 4 and Figure 3C,D show the alignment between the experimental and predicted pIC_50_ values for the CoMFA and CoMSIA models. Considering the agreement between the experimental and predicted biological activity for the test set, the 3D QSAR models have a suitable ability to predict the activity of novel compounds belonging to the structural class studied.

### 3.3. Applicability Domain

**Figure 4:**
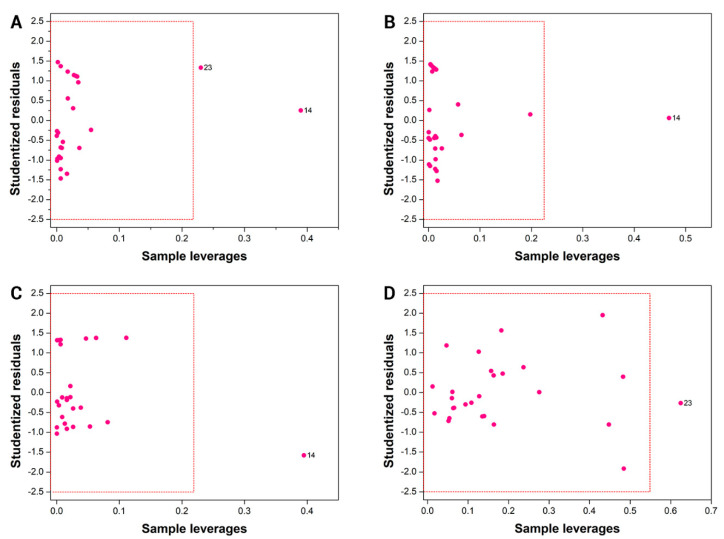
Applicability domain for the final QSAR models. (**A**) AutoQSAR; (**B**) HQSAR; (**C**) CoMFA; and (**D**) CoMSIA.

The leverage and Student residuals are complementary indicators that can be used to detect structural and activity outliers [38]. The leverage measures the influence of a sample on the construction of a QSAR model. It can be regarded as the distance of a compound to the center of the training set in a space that is defined by the molecular descriptors. Student residues are defined in units of standard deviation from the mean value. Values greater than ±2.5 are considered outside the usual statistical conditions and define compounds that are called activity outliers. Compounds that have a high leverage on the training of the model are called structural outliers. Figure 4 indicates that compounds 14 and 23 had leverage values above the established limit, and can be considered structural outliers, i.e., compounds that had a high influence on the construction of the models. These two molecules have an aromatic ring attached to the imidazole ring, which is a distinguishing feature with respect to the other compounds in the training set. This structural feature is probably the cause of the high leverage of compounds 14 and 23 on the models. Importantly, no activity outliers were detected, which demonstrates the predictive power of the models.

### 3.4. 2D Contribution Maps

**Figure 5:**
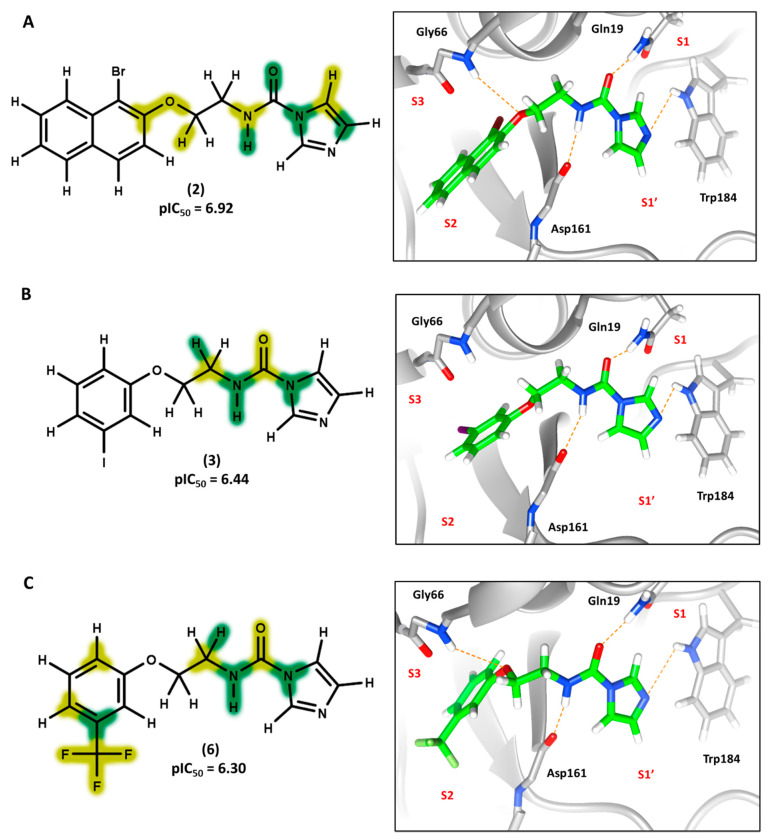
Contribution maps showing the key role played by the imidazole, linker, and aryl halide fragments in activity and binding conformations predicted by molecular docking. (**A**) Compound 2; (**B**) compound 3; (**C**) compound 6. Inhibitors and active site residues are depicted as sticks, and hydrogen bonds are shown as dashed lines. Contribution maps: Yellow and green represent positive contributions, while red, orange, and red-orange indicate negative contributions to activity. White represents intermediate contributions.

**Figure 6:**
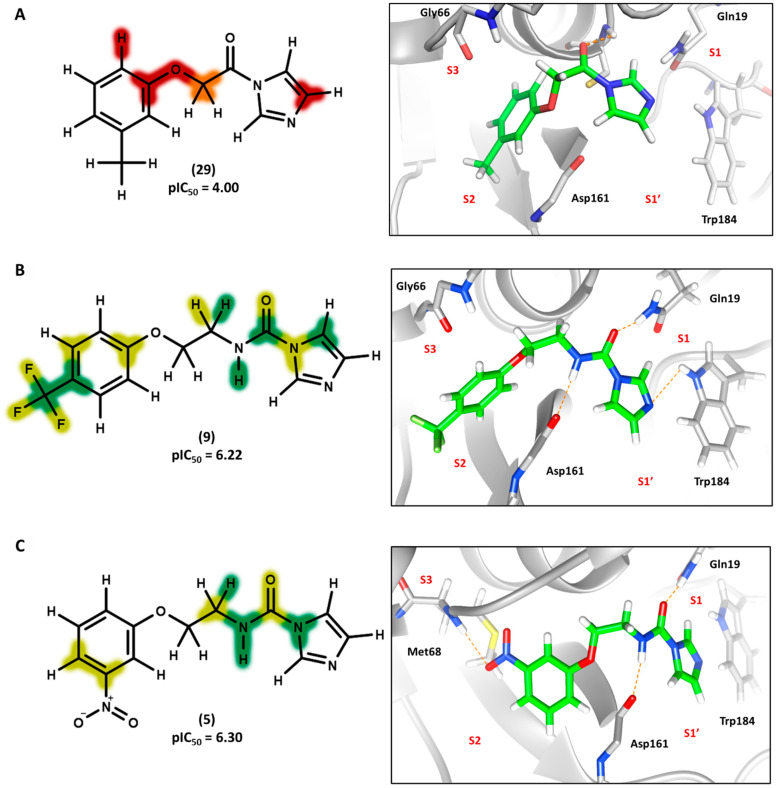
Contribution maps showing the influence of the imidazole, linker and aryl substituents on activity and binding conformations predicted by molecular docking. (**A**) Compound 29; (**B**) compound 9; (**C**) compound 5. Inhibitors and active site residues are depicted as sticks, and hydrogen bonds are shown as dashed lines. Contribution maps: Yellow and green represent positive contributions, while red, orange, and red-orange indicate negative contributions to activity. White represents intermediate contributions.

The best HQSAR model was used to create color-coded 2D maps that assigned negative and positive contributions to activity for each molecular fragment. Figure 5 shows the contribution maps for the highly potent compounds 2, 3, and 6 along with their predicted binding conformations. The contribution maps indicate intermediate and positive contributions of aryl halide groups to the biological activity, corroborated by the binding mode of inhibitors 2, 3, and 6 with cruzain. The aryl halide rings of these compounds interact with the S2 subsite, which consists predominantly of hydrophobic residues. In the linker that connects the two ring systems, the amide and ether groups were flagged, playing an important role in enzyme inhibition. Indeed, the predicted binding conformations for compounds 2, 3, and 6 show the ether oxygen interacting with Gly66 and the amide hydrogen and oxygen forming hydrogen bonds with Asp161 and Gln19, respectively. The importance of the linker was further demonstrated by the negative contributions attributed to compound 29 (Figure 6A). This compound had the linker shortened by the removal of the amide nitrogen and one of the methylene groups, which radically decreased its potency. This reduction in activity probably occurred because of the loss of key hydrogen bonds with Gln19 and Asp161. Additionally, the shortening of the linker hindered the proper positioning of the two rings in the S1′ and S2 subsites, which hampered the key elements that support cruzain-inhibitor binding.

Keeping the optimum linker configuration and varying the substitution pattern at the phenyl ring revealed further intermediate and positive contributions to activity (Figure 6B,C). The molecular docking results for compound 9 (Figure 6B) confirmed that hydrophobic moieties enable full interaction with the S2 subsite and represent an important driver of enzyme-inhibitor interactions. Otherwise, the polar nitro group of inhibitor 5 (Figure 6C) allows the phenyl ring to access the S2 subsite by establishing a hydrogen bond with Met68, demonstrating that specific polar interactions can also mediate a proper interaction with the S2 subsite.

**Figure 7:**
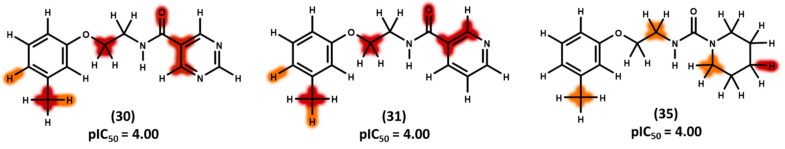
Contribution maps for compounds that had the imidazole ring replaced by other rings, highlighting the detrimental effect on the activity against cruzain.

For the imidazole ring, most compounds in the dataset exhibited positive contributions for this group (Figure 5 and Figure 6). Imidazole was found to interact with Trp184, which plays an important role in anchoring the inhibitors at the solvent-exposed S1/S1′ interface. Replacing the imidazole with rings such as pyrimidine, pyridine, and piperidine severely decreased activity, as shown by the generalized negative contributions shown in Figure 7.

### 3.5. 3D Contour Maps

**Figure 8:**
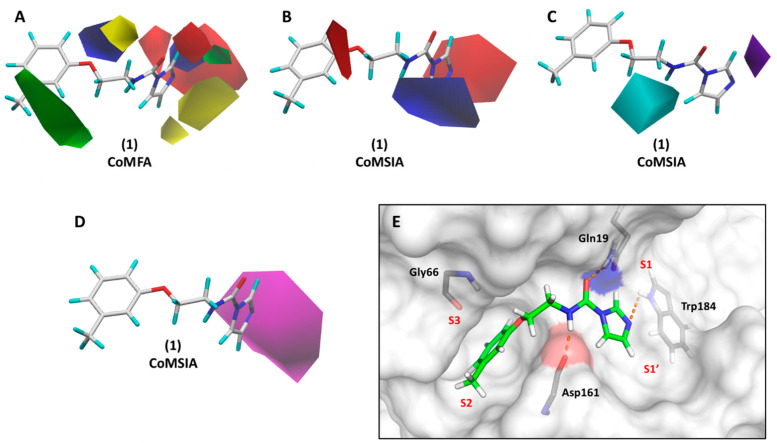
3D StDev*Coeff CoMFA and CoMSIA contour maps for compound 1. (**A**) CoMFA steric and electrostatic contour maps; (**B**) CoMSIA electrostatic contour maps; (**C**) CoMSIA hydrogen-bond donor contour maps; (**D**) CoMSIA hydrogen-bond acceptor contour maps; (**E**) predicted binding conformation of compound 1. Compound 1 and cruzain binding site residues are depicted as sticks. Cruzain is shown as a surface, with Asp161 highlighted in red and Gln19 highlighted in blue. Hydrogen bonds are shown as dashed lines. Green and yellow plots: Bulky groups are favorable and unfavorable to activity, respectively. Blue and red contours: Positive and negative groups, respectively, are favorable to activity. Cyan and purple plots: Hydrogen bond donors are favorable and unfavorable to activity, respectively. Magenta: Hydrogen-bond acceptors are favorable to activity.

3D QSAR contour maps were used to assign the leverage of steric, electrostatic, and hydrogen-bond donor/acceptor features on the activity against cruzain. CoMFA and CoMSIA green steric maps designate areas where bulky groups are associated with increased biological activity. Yellow contours, otherwise, indicate regions where the introduction of bulky moieties correlates with reduced activity. Regarding the electrostatic maps, red and blue contours designate areas where the increment in negative and positive charge, respectively, correlates with increased biological activity. Figure 8 depicts CoMFA and CoMSIA contour maps for the lead compound (1, pIC_50_ = 6.00). Figure 8A shows the phenyl ring surrounded by green contours, which is aligned with the role played by bulky groups in the interaction with the S2 subsite, a feature that was also highlighted by the 2D contribution maps (Figure 5 and Figure 6). Yellow plots around the linker support the concept that the introduction of bulky substituents in this region is detrimental to activity. Bulky groups could disrupt the hydrogen bonds between the linker and Gln19 and Asp161. These residues border a relatively narrow channel that connects the two largest cavities formed by the S1′ and S2 subsites (Figure 8E). Additionally, the electrostatic maps show red areas surrounding the amide oxygen and the imidazole nitrogen, highlighting the importance of negative dipoles in these regions. These findings are supported by the interaction of the amide oxygen and imidazole nitrogen with Gln19 and Trp184, respectively. Moreover, the blue maps next to the amide and imidazole hydrogens suggest that increasing the positive charge at these areas could enhance the activity of the compounds.

The CoMSIA electrostatic maps (Figure 8B) reinforce the favorable role played by the negative dipole at the imidazole 3-nitrogen and the positive partial charge at the amide and imidazole hydrogen atoms. Furthermore, the CoMSIA electrostatic maps stress the relevance of the negative partial charge next to the phenyl ring, as indicated by the red contour surrounding the ether oxygen. The ether oxygen of several compounds was predicted to interact with Gly66 at the S3 subsite (Figure 5). CoMSIA hydrogen bond donor and acceptor contour plots are pictured in Figure 8C,D, respectively. Cyan and purple spots near the amide hydrogen and imidazole 3-nitrogen show that hydrogen bond donors are favorable and detrimental to biological activity, respectively. This is consistent with the binding mode of compound 1 (Figure 8E), which shows a hydrogen bond between the amide hydrogen and Asp161 and the imidazole 3-nitrogen acting as an acceptor. The importance of the hydrogen-bond acceptor characteristic of imidazole is additionally stressed by the magenta contour shown in Figure 8D. The interaction of the imidazole 3-nitrogen with Trp184 was revealed to be highly relevant to cruzain inhibition. This interaction can be responsible for stabilizing the imidazole group at the large and solvent-exposed S1/S1′ interface, providing a key enthalpic drive for cruzain-inhibitor binding. Overall, the 3D QSAR contour maps highlight molecular attributes that closely correlate with the 2D QSAR contribution maps. Altogether, both the 2D and 3D QSAR models and the molecular docking results were able to disclose relevant aspects underlying cruzain inhibition and the enzyme–inhibitor interaction.

The development of QSAR models has been a valuable tool to study cruzain inhibitors, and broadly diverse techniques have been reported recently. In a 3D QSAR study, Saraiva and co-workers developed CoMFA and CoMSIA models for a series of α–keto-based cruzain inhibitors. Solid models were built with *r*^2^_pred_ = 0.72 for CoMFA and 0.97 for CoMSIA. Additionally, molecular dynamics simulations were carried to examine the binding mode of the inhibitors [39]. Pauli et al. developed HQSAR, CoMFA, and CoMSIA models for a series of benzimidazole derivatives. The final models showed sound statistical parameters and proved to be predictive for the test set compounds with *r*^2^_pred_ of 0.65, 0.94, and 0.82 for HQSAR, CoMFA, and CoMSIA, respectively [21]. In another study, Scotti and colleagues used multiple linear regression (MLR), the best-first algorithm, and PLS to generate QSAR models (*r*^2^_ext_ = 0.79) for 61 semicarbazones. The variable selection approach used in the study resulted in the extraction of a few 1D, 2D, and 3D descriptors from more than 4800 attributes [40]. Structurally diverse cruzain inhibitors were investigated by Rosas–Jimenez and co-workers. More than 800 cruzain inhibitors were collected from the literature and QSAR models were built using the k-nearest neighbors and random forest techniques. 1D and 2D descriptors were used and the external predictive ability of the models was demonstrated by *r*^2^_ext_ values of 0.72 and 0.76 [41].

As one can see from studies that have been conducted recently, different QSAR approaches can be used to produce statistically sound models with good predictive ability. Our models are comparable with these studies in terms of statistical robustness and predictive power. Furthermore, the QSAR effort described here can contribute to the advance of the field regarding the following aspects. In this work, we report QSAR models for reversible, competitive, and non-peptidic cruzain inhibitors belonging to the imidazole class. These compounds showed promising properties including in vivo efficacy against an animal model of Chagas disease and no acute toxicity. In addition, the initial hit was discovered in a virtual screening and the analogues were optimized by extensive computational, synthetic, and biological studies entirely conducted in our laboratory.

Although the QSAR models described here were able to predict accurately the activity of the test set compounds and identify features that are determinant for cruzain inhibition, every QSAR method has advantages and limitations. 2D techniques such as AutoQSAR and HQSAR have the important advantage of being independent of molecular alignment, which makes these methods very straightforward. This is an important feature as these techniques require significantly less time for model development. Both 2D methods are based on molecular fragments as descriptors, and this type of independent variable has been widely used [42]. Besides molecular fragmentation, AutoQSAR computes physicochemical properties, which significantly expands the scope of molecular descriptors considered in the construction of the models in comparison with HQSAR. However, 2D methods have important limitations. The main disadvantage is that three-dimensional properties that determine the interaction with molecular targets are not considered. No insight into ligand–receptor interaction has been provided by taking the output of 2D methods alone.

Otherwise, 3D methods such as CoMFA and CoMSIA allow the interpretation of the results in terms of molecular features that are closely related to ligand–receptor interactions. Features such as electrostatic and steric potentials and hydrogen-bonding ability are especially useful to presume which type of intermolecular interactions can take place during the formation of the ligand–receptor complex. When the structure of the molecular target is known, it can be integrated to the output of 3D QSAR models to give a much more detailed picture of ligand–receptor molecular recognition. An important disadvantage of 3D methods is the dependence on structural alignment, which can significantly slow the process of model development. Additionally, defining a method for structural alignment is not straightforward, mainly when the molecular target is unknown. One key difference in the output of the 3D methods used here is that CoMFA maps are more fragmented and apart from the ligand surfaces than CoMSIA maps [20]. This occurs because of the cutoff parameterization of the CoMFA fields which results in a high steepness of the electrostatic and steric potentials when the probe comes too close to the molecular surfaces. Otherwise, the Gaussian function implemented in CoMSIA is significantly softer than the Lennard–Jones and Coulomb functions used in CoMFA. The use of the CoMSIA Gaussian function facilitates the interpretation of the contour maps as they become more continuous and able to fill in the region occupied by the compounds.

## 4. Conclusions

A series of cruzain inhibitors featuring an imidazole core was used to develop predictive 2D and 3D QSAR models. The best models resulting from each QSAR technique—HQSAR, AutoQSAR, CoMFA, and CoMSIA—showed sound statistical consistency and high predictive power for untested compounds. The QSAR graphical output was examined along with molecular docking results, which enabled the main findings of both ligand- and structure-based approaches to be integrated. This complementary strategy uncovered pivotal 2D and 3D properties that strongly affected the activity of the dataset compounds. The imidazole core interacted with Trp184 and proved to be essential for activity. This finding demonstrates the importance of establishing polar contacts at the solvent-exposed S1/S1′ interface. A linker with a length of five atoms with two hydrogen bond acceptors and one donor proved to be ideal. This linker design allowed the two ring systems at the molecular ends to be positioned properly and interact optimally with the S1′ and S2 cavities. Finally, ligand- and structure-based studies highlighted the essential role played by bulky groups in fulfilling the S2 subsite. Altogether, the results reported herein revealed significant molecular aspects that drive cruzain–inhibitor recognition. These findings can guide further drug discovery campaigns aimed at the design of cruzain inhibitors that can lead to novel and effective drug candidates for Chagas disease.

## Figures and Tables

**Table 1:** Structure of the dataset compounds and pIC_50_ values.

Inhibitor	Structure	pIC_50_^a^
1	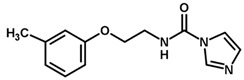	6.00
2 *	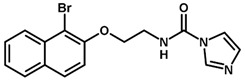	6.92
3	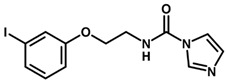	6.44
4 *	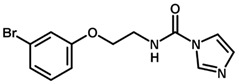	6.39
5	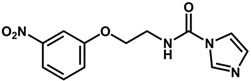	6.30
6	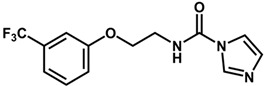	6.30
7	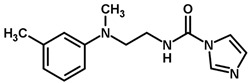	6.28
8	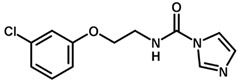	6.24
9 *	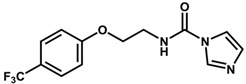	6.22
10	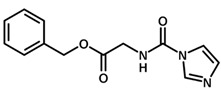	6.22
11	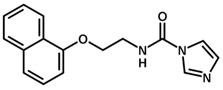	6.18
12 *	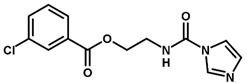	6.15
13	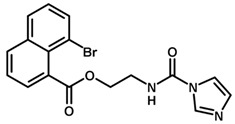	6.12
14	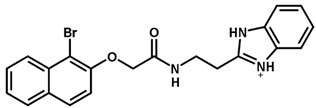	6.68
15	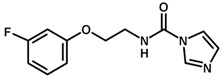	5.77
16 *	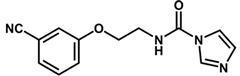	5.74
17	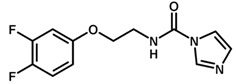	5.74
18	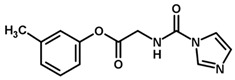	5.70
19	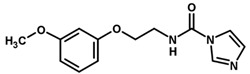	5.60
20	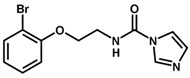	5.55
21 *	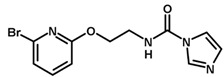	5.52
22	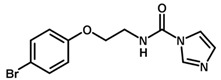	5.51
23	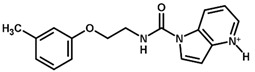	5.49
24	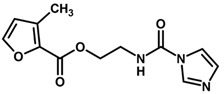	5.49
25 *	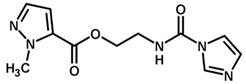	5.32
26	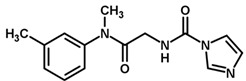	5.21
27 *	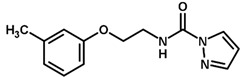	5.21
28	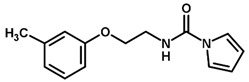	4.11
29	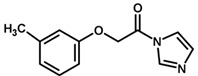	4.00
30 *	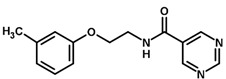	4.00
31	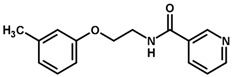	4.00
32	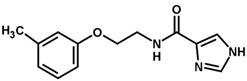	4.00
33	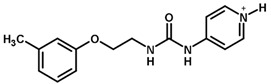	4.00
34	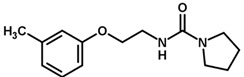	4.00
35	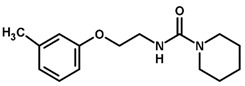	4.00
36 *	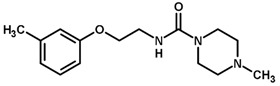	4.00
37	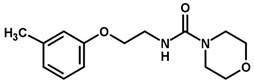	4.00

^a^ pIC_50_ (−log IC_50_); * test set compounds.

**Table 2:** Hologram quantitative structure–activity relationship (HQSAR) models obtained by using fragment sizes varying from 4 to 7 atoms.

Model	Fragment Distinction	*q* ^2^	*r* ^2^	SEE	HL	N
1	A/B/C	0.70	0.92	0.28	199	3
2	A/B/C/Ch	0.70	0.92	0.28	199	3
3	A/B/C/H	0.72	0.90	0.33	83	4
4	A/B/C/H/Ch	0.72	0.90	0.33	83	4

A = atoms; B = bonds; C = connectivity; H = hydrogen atoms; Ch = chirality; *q*^2^ = leave-one-out (LOO) cross-validated correlation coefficient; *r*^2^ = non-cross-validated correlation coefficient; SEE = standard error of estimate; HL = hologram length; N = optimum number of components.

**Table 3:** Best HQSAR models obtained by varying fragment size.

Model	Fragment Distinction	Fragment Size	*q* ^2^	*r* ^2^	*r* ^2^_pred_	SEE	HL	N
5	A/B/C	2–7	0.70	0.92	0.78	0.27	199	3
6	A/B/C	3–7	0.71	0.92	0.80	0.28	199	3
8	A/B/C/H	2–6	0.76	0.90	0.67	0.33	83	4
7	A/B/C/Ch	3–7	0.70	0.92	0.78	0.28	199	3
9	A/B/C/H/Ch	2–6	0.76	0.90	0.67	0.33	83	4

A = atoms; B = bonds; C = connectivity; H = hydrogen atoms; Ch = chirality; *q*^2^ = leave-one-out (LOO) cross-validated correlation coefficient; *r*^2^ = non-cross-validated correlation coefficient; *r*^2^_pred_ = predictive correlation coefficient; SEE = standard error of estimate; HL = hologram length; N = optimum number of components.

**Table 4:** Experimental and predicted pIC_50_ values for the final HQSAR, AutoQSAR, comparative molecular field analysis (CoMFA), and comparative molecular similarity indices analysis (CoMSIA) models.

Inhibitor	Experimental	HQSAR		AutoQSAR		CoMFA		CoMSIA	
		Predicted	Residual ^1^	Predicted	Residual ^1^	Predicted	Residual ^1^	Predicted	Residual ^1^
1	6.00	5.34	0.66	5.49	0.51	5.78	0.22	5.85	0.15
2 *	6.92	6.25	0.67	6.38	0.54	6.08	0.84	6.27	0.65
3	6.44	6.02	0.42	6.27	0.17	6.47	−0.03	6.46	−0.02
4 *	6.39	5.96	0.43	5.98	0.41	6.01	0.38	6.05	0.34
5	6.30	6.14	0.16	6.24	0.06	6.32	−0.02	6.01	0.29
6	6.30	6.49	−0.19	6.30	0.00	6.42	−0.12	6.08	0.22
7	6.28	5.93	0.35	5.85	0.43	6.28	0.00	6.21	0.07
8	6.24	5.90	0.34	6.23	0.01	6.25	−0.01	6.02	0.22
9 *	6.22	6.60	−0.38	6.11	0.11	6.04	0.18	5.93	0.29
10	6.22	5.99	0.23	6.04	0.18	6.19	0.03	6.16	0.06
11	6.18	6.07	0.11	6.36	−0.18	6.17	0.01	6.09	0.09
12 *	6.15	5.65	0.50	5.99	0.16	6.04	0.11	6.16	−0.01
13	6.12	5.93	0.19	5.95	0.17	6.12	0.00	6.13	−0.01
14	6.68	6.90	−0.22	6.06	0.62	6.67	0.01	6.53	0.15
15	5.77	5.83	−0.06	6.19	−0.42	5.68	0.09	5.75	0.02
16 *	5.74	5.81	−0.07	6.10	−0.36	6.10	−0.36	6.04	−0.30
17	5.74	5.93	−0.19	6.04	−0.30	5.79	−0.05	5.79	−0.05
18	5.70	5.91	−0.21	5.53	0.17	5.71	−0.01	5.79	−0.09
19	5.60	5.80	−0.20	5.99	−0.39	5.67	−0.07	5.80	−0.20
20	5.55	5.92	−0.37	5.76	−0.21	5.61	−0.06	6.03	−0.48
21 *	5.52	5.82	−0.30	5.85	−0.33	5.55	−0.03	5.87	−0.35
22	5.51	5.93	−0.42	5.78	−0.27	5.56	−0.05	5.85	−0.34
23	5.49	5.63	−0.14	5.14	0.35	5.54	−0.05	5.66	−0.17
24	5.49	5.44	0.05	5.52	−0.03	5.39	0.10	5.62	−0.13
25 *	5.32	5.60	−0.28	5.56	−0.24	5.85	−0.53	5.88	−0.56
26	5.21	5.39	−0.18	5.39	−0.18	5.11	0.10	4.87	0.34
27 *	5.21	4.49	0.72	4.99	0.22	4.64	0.57	4.26	0.95
28	4.11	4.54	−0.43	4.80	−0.69	4.23	−0.12	4.31	−0.20
29	4.00	4.21	−0.21	4.47	−0.47	3.98	0.02	3.99	0.01
30 *	4.00	3.90	0.10	4.07	−0.07	3.91	0.09	4.18	−0.18
31	4.00	3.89	0.11	3.99	0.01	4.03	−0.03	3.81	0.19
32	4.00	4.08	−0.08	3.95	0.05	3.98	0.02	3.95	0.05
33	4.00	3.85	0.15	4.17	−0.17	3.94	0.06	4.06	−0.06
34	4.00	3.95	0.05	3.79	0.21	4.04	−0.04	4.23	−0.23
35	4.00	3.98	0.02	3.79	0.21	3.96	0.04	3.82	0.18
36 *	4.00	3.95	0.05	3.96	0.04	3.80	0.20	4.55	−0.55
37	4.00	4.01	−0.01	3.87	0.13	4.06	−0.06	4.08	−0.08

^1^ Difference between experimental and predicted pIC_50_; * test set compounds.

**Table 5:** Best models generated by AutoQSAR.

Training Set (%)	Score	*r* ^2^	SD	*q* ^2^	RMSE	N	Fingerprint
70	0.85	0.85	0.40	0.82	0.37	2	Desc
72	0.90	0.89	0.32	0.90	0.29	2	Dendritic
74	0.82	0.82	0.44	0.78	0.39	2	Desc
76	0.81	0.81	0.43	0.78	0.40	2	Desc
78	0.81	0.81	0.43	0.78	0.40	2	Desc
80	0.82	0.82	0.43	0.91	0.26	2	Desc

Score: Performance considering training and test set predictive accuracies; *r*^2^: Coefficient of determination for the training set; SD: Standard deviation; *q*^2^: Predictive correlation coefficient for the test set (*r*²_pred_); RMSE: Root mean square error for the test set predictions; N: Optimum number of components.

**Table 6:** Final CoMFA and CoMSIA models derived by applying different StDev*Coeff and grid spacing values.

Model	*q* ^2^	*r* ^2^	*r* ^2^_pred_	SEE	N	F	S	E	H	D	A
CoMFA	0.72	0.99	0.81	0.08	6	595.85	0.41	0.59	-	-	-
CoMSIA	0.63	0.96	0.73	0.21	3	179.60	0.11	0.34	0.17	0.14	0.24

*q*^2^: Cross-validated correlation coefficient; *r*^2^: Non-cross-validated correlation coefficient; *r*^2^_pred_: Predictive correlation coefficient; SEE: Standard error of estimate; N: Optimum number of components; F: F-test; S: Steric fraction; E: Electrostatic fraction; H: Hydrophobic fraction; D: Donor fraction; A: Acceptor fraction.
